# Prompt access to effective malaria treatment among children under five in sub-Saharan Africa: a multi-country analysis of national household survey data

**DOI:** 10.1186/s12936-015-0844-7

**Published:** 2015-08-25

**Authors:** Jui A. Shah, Jacques B. O. Emina, Erin Eckert, Yazoume Ye

**Affiliations:** MEASURE Evaluation/ICF International, Rockville, MD USA; Department of Population and Development Studies, University of Kinshasa, Kinshasa, Democratic Republic of Congo; President’s Malaria Initiative, US Agency for International Development, Washington, DC, USA

**Keywords:** Malaria treatment, Prompt treatment, Under-five children, SSA, Malaria-endemic country, Anti-malarial, ACT

## Abstract

**Background:**

Scaling up diagnostic testing and treatment is a key strategy to reduce the burden of malaria. Delays in accessing treatment can have fatal consequences; however, few studies have systematically assessed these delays among children under five years of age in malaria-endemic countries of sub-Saharan Africa. This study identifies predictors of prompt treatment with first-line artemisinin combination therapy (ACT) and describes profiles of children who received this recommended treatment.

**Methods:**

This study uses data from the most recent Demographic and Health Survey, Malaria Indicator Survey, or Anaemia and Parasite Prevalence Survey conducted in 13 countries. A Chi square automatic interaction detector (CHAID) model was used to identify factors associated with prompt and effective treatment among children under five years of age.

**Results:**

The percentage of children with fever who received any anti-malarial treatment varies from 3.6 % (95 % CI 2.8–4.4 %) in Ethiopia to 64.5 % (95 % CI 62.7–66.2 %) in Uganda. Among those who received prompt treatment with any anti-malarial medicine, the percentage who received ACT ranged from 32.2 % (95 % CI 26.1–38.4 %) in Zambia to nearly 100 % in Tanzania mainland and Zanzibar. The CHAID analysis revealed that country of residence is the best predictor of prompt and effective treatment (p < 0.001). Depending on the country, the second best predictor was maternal education (p = 0.004), place of residence (p = 0.008), or household wealth index (p < 0.001).

**Conclusions:**

This study reveals that country of residence, maternal education, place of residence, and socio-economic status are key predictors of prompt access to malaria treatment. Achieving universal coverage and the elimination agenda will require effective monitoring to detect disparities early and sustained investments in routine data collection and policy formulation.

## Background

Malaria burden remains high in sub-Saharan Africa (SSA) and affects mostly children under five years of age [1]. Several strategies have been proposed to reduce this burden, including improved vector control, intermittent preventive treatment during pregnancy, and standardized case management. For these interventions to be most effective, however, they must reach national and global coverage targets [2].

The World Health Organization (WHO) recommends prompt treatment, usually within 24 h of the onset of fever, with recommended anti-malarial medicines after confirmation of malaria through appropriate diagnostic tests [1]. Timely access to effective treatment with first-line artemisinin combination therapy (ACT) is fundamental to preventing progression of *Plasmodium falciparum* malaria to severe malaria and death, particularly among children under five years of age. Across SSA, only a small proportion of malaria patients, including children, receive prompt and effective treatment [1]. In Kenya, a systematic review documented a delay in treatment for fever, with only 5 % of fever cases receiving prompt treatment with an anti-malarial drug [3]. Similarly, in Tanzania, Khatib and colleagues found that less than 50 % of fever cases received treatment with ACT within 24 h [4]. Potential reasons for low access to prompt and effective treatment among children revolve around affordability, acceptability, availability, and adequacy to meet expectations in quality of care [5–9].

Several studies have looked into a specific context, dimension, or population related to prompt and effective treatment for malaria; however, few studies systematically look across all children in SSA [10, 11]. This study aims to fill this gap by examining the extent to which children under five years of age in selected countries in SSA received prompt and effective treatment and describing profiles of children who received this recommended treatment. The study also identifies subgroups that, when successfully targeted, may optimize benefits of interventions within the entire population. Although no longer recommended as a key malaria control indicator due to challenges in its interpretation [12], ‘promptness’ was assessed by whether treatment commenced the same day or day after onset of fever. The work involves the following US President’s Malaria Initiative (PMI) focus countries that have relevant national survey data and that were selected for impact evaluations: Angola, Benin, Ethiopia, Ghana, Kenya, Liberia, Madagascar, Malawi, Mali, Mozambique, Rwanda, Senegal, United Republic of Tanzania (mainland and Zanzibar), Uganda, and Zambia.

## Methods

### Data used for the analysis

**Fig. 1:**
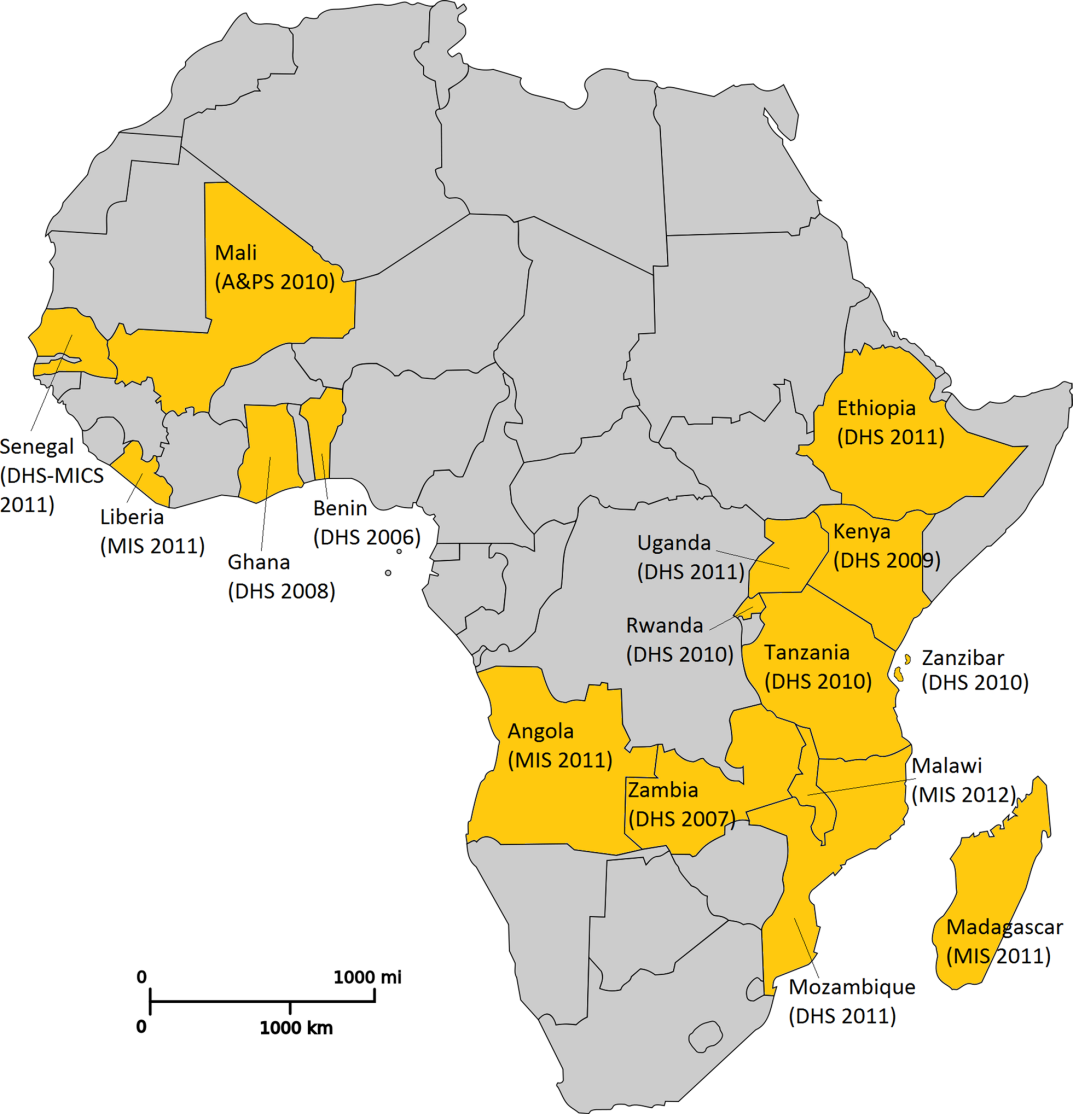
Countries and household surveys included in the study

This study uses data from the most recent Demographic and Health Survey (DHS), Malaria Indicator Survey (MIS), or Anaemia and Parasite Prevalence Survey (A&PS) conducted in each PMI priority country. Each of these surveys included a malaria module, in addition to the standard household and women’s questionnaires. All surveys were conducted between 2006 (Benin) and 2012 (Malawi), with most carried out in 2010 and 2011. The countries belong to three geographical regions of SSA: (1) west, (2) central, and (3) east (Fig. 1).

### Anti-malarial drug policies

All countries had adopted ACT as first-line treatment for malaria (‘effective treatment’) by the time the surveys were conducted; however, the duration between policy adoption and the survey varies from 2 years (Benin) to 7 years (Liberia, Ethiopia, and Mozambique), with an average of 5.2 years (Table 1).

**Table 1:** Duration between year of ACT policy adoption and implementation of surveys

Country	Type of ACT	Year adopted	Year of survey	Time in years between policy adoption and survey
Angola	ALu	2006	2011	5
Benin	ALu	2004	2006	2
Ethiopia	ALu	2004	2011	7
Ghana	ASAQ, ALu	2004	2008	4
Kenya	ALu	2004	2009	5
Liberia	ALu	2004	2011	7
Madagascar	ASAQ	2006	2011	5
Malawi	ASAQ, ALu	2007	2012	5
Mali	ASAQ, ALu	2007	2010	3
Mozambique	ALu	2004	2011	7
Rwanda	ALu	2005	2010	5
Senegal	ASAQ	2005	2011	5
Tanzania mainland	ALu, ASAQ	2004	2010	6
Uganda	ALu	2004	2011	6
Zambia	ALu	2002	2007	5
Zanzibar	ASAQ	2004	2010	6

*ALu* artemether–lumefantrine, *ASAQ* arthesunate–amodiaquine

### Data quality checks

Data quality checks included assessing response rates, evaluating completeness of data for key variables, and assessing reliability of birth history data. Response rates refer to the percentage of the number of people or households in the sample that completed an interview. In each country, more than 90 % of identified households and women were successfully interviewed, confirming adequate response rates. Misreporting of ages or dates of birth can affect malaria estimates because malaria infection varies significantly by a child’s age [13, 14]. In each country included in the study, at least 93 % of children had complete month and year of birth data, so no country was eliminated due to incomplete information. The reliability of maternal reporting was assessed by analysing the number of live births per year. Overall, the number of births was high for the 4 years preceding the survey but lower in the fifth year preceding the survey. This distribution did not, however, affect the overall reliability of the birth history data.

Of 16 countries, 14 had information on whether anti-malarial treatment was received on the same or next day as the onset of fever (‘prompt treatment’). Two countries, Ethiopia and Mali, were excluded from the bivariate and multivariate analyses because they do not include this information. In addition, the Benin database did not have time-to-treatment for ACT and was excluded from some analyses.

### Statistical analysis

The analysis included two steps. The first step involved computing proportions and conducting Chi square tests for each country to identify associations between prompt treatment and selected background characteristics of children, their primary caretakers, and their households. The data was weighted to account for oversampling, undersampling, and varying response in different regions included in national household surveys. The second step consisted of pooling data from all countries and running a Chi square automatic interaction detector (CHAID) model [15, 16]. This method is a sequential fitting algorithm; at each step, the model chooses the predictor variable that has the strongest interaction with an outcome of interest: here, prompt and effective treatment. The variable with the strongest association becomes the first branch of the tree, with a leaf for each category that is significantly different. CHAID then assesses the category groupings to pick the most significant combination of variables. It is particularly useful for identifying sub-groups that are more or less likely to experience the outcome of interest and for determining the relative contributions of these groups to overall coverage in the general population.

The analysis was restricted to children with fever who received any anti-malarial medicine. The covariates were the child’s sex, age group in months (<6, 6–23, 24–59), relationship to the head of household (child or stepchild, grandchild, other), maternal age in years (15–19, 20–29, 30–49), maternal education (none, primary, secondary and above), place of residence (urban, rural), household wealth quintiles, and country of residence.

The output of the CHAID model is presented in a hierarchical tree structure and consists of several levels of branches: root node, parent nodes, child nodes, and terminal nodes. The root node, ‘Node 0’, comprises children who received any anti-malarial medicine. Parent nodes are upper nodes compared to lower-level child nodes. Terminal nodes are any node that does not have child nodes. For each terminal node, the CHAID model provides the following indicators: (1) demographic weight in the sample; (2) gain, the number of children who received prompt and effective treatment in the terminal node, divided by the total number of children who received any anti-malarial treatment; (3) response, the proportion of children who received prompt and effective treatment among all those within the terminal node; and, (4) gain index percentage, which represents the increased probability of prompt and effective treatment in the terminal node compared to the overall study population.

## Results

### Treatment of fever among children under five

Among children with fever, the proportion that received any anti-malarial treatment varied from 3.6 % (95 % CI 2.8–4.4 %) in Ethiopia to 64.5 % (95 % CI 62.7–66.2 %) in Uganda. Coverage was above 50 % in four countries: Benin, Liberia, Tanzania mainland, and Uganda. The percentage of children with fever who received an ACT was below 10 % in nearly half of the countries included in the analysis. Higher percentages of children received an ACT in Uganda (44.2 %, 95 % CI 42.4–46.1 %), Liberia (39.7 %, 95 % CI 37.3–42.1 %), and Tanzania mainland (37.9 %, 95 % CI 35.1–40.6 %). Coverage was particularly low in Benin (0.9 %, 95 % CI 0.6–1.1 %), where ACT was rolled out in 2004, just 2 years before the survey [17]. The percentage of children who received ACT, among those who received any anti-malarial treatment, varied from 1.6 % (95 % CI 1.1–2.1 %) in Benin to 95.7 % (95 % CI 92.3–99.1 %) in Rwanda (Table 2).

**Table 2:** Percentage of children under five with fever in the 2 weeks before the survey who received treatment with anti-malarial drug by country

Country	Children with fever in the 2 weeks before the survey			Children who received ACT among those who received any anti-malarial treatment		
	Received any anti-malarial	Received ACT		% (95% CI)	N	Year
	% (95% CI)	% (95% CI)	N			
Angola	28.3 (26.6–30.0)	21.7 (20.1–23.2)	2645	76.6 (73.5–79.6)	738	2011
Benin^a^	54.0 (52.5–55.5)	0.9 (0.6–1.1)	4204	1.6 (1.1–2.1)	2243	2006
Ethiopia	3.6 (2.8–4.4)	1.3 (0.8–1.8)	2082	36.3 (27.8–44.9)	124	2011
Ghana	43.0 (38.8–47.1)	21.5 (18.1–24.9)	551	50.0 (44.5–56.6)	225	2008
Kenya	23.2 (20.9–25.4)	7.8 (6.3–9.2)	1385	33.5 (28.2–38.7)	311	2009
Liberia	57.1 (54.7–59.5)	39.7 (37.3–42.1)	1617	69.6 (66.5–72.6)	895	2011
Madagascar	19.8 (17.3–22.4)	3.8 (2.6–5.0)	959	19.3 (13.4–25.1)	177	2011
Malawi	32.5 (29.0–36.0)	29.6 (26.1–33.0)	676	91.0 (87.1–94.8)	216	2012
Mali	34.7 (31.2–38.2)	7.8 (5.8–9.7)	705	22.4 (17.1–27.6)	243	2010
Mozambique	29.9 (27.4–32.4)	17.9 (15.8–20.0)	1313	59.9 (54.9–64.9)	366	2011
Rwanda	10.8 (9.2–12.5)	10.4 (8.7–12.0)	1332	95.7 (92.3–99.1)	140	2010
Senegal	8.2 (7.1–9.3)	3.4 (2.6–4.1)	2314	41.0 (33.7–48.3)	176	2010
Tanzania mainland	60.1 (57.4–62.7)	37.9 (35.1–40.6)	1320	63.1 (59.7–66.5)	785	2010
Uganda	64.5 (62.7–66.2)	44.2 (42.4–46.1)	2860	68.6 (66.5–70.7)	1849	2010
Zambia	38.4 (35.4–41.3)	11.1 (9.2–13.0)	1034	29.0 (24.6–33.4)	417	2007
Zanzibar	16.9 (12.5–21.3)	5.6 (2.9–8.3)	282	33.1 (18.3–48.0)	42	2010

*95* *% CI* 95 % confidence interval, *N* number of children

^a^Artemisinin combination therapy [ACT] was rolled out in Benin only 2 years before the survey

### Prompt treatment of fever among children under five

The percentage of children who received prompt treatment, among children who received any anti-malarial drug, varied from 42.1 % (95 % CI 34.8–49.4 %) in Madagascar to 87.2 % (95 % CI 76.7–97.8 %) in Zanzibar. Among those who received prompt treatment with any anti-malarial medicine, the percentage who received ACT ranged from 32.2 % (95 % CI 26.1–38.4 %) in Zambia to nearly 100 % in Tanzania mainland and Zanzibar (Table 3).

**Table 3:** Percentage of children under five with fever in the 2 weeks before the survey who received prompt treatment with anti-malarial drug by country

Country	Prompt treatment with any anti-malarial drug among those who received any anti-malarial drug		Prompt treatment with ACT among those who received prompt treatment with any anti-malarial drug	
	% (95% CI)	N^a^	% (95% CI)	N^a^
Angola	57.8 (51.2–61.4)	738	72.4 (68.0–76.8)	398
Benin^b^	77.8 (76.0–79.5)	2243	n/a	n/a
Ethiopia^c^	n/a	n/a	n/a	n/a
Ghana	55.2 (48.6–61.7)	225	51.0 (42.0–59.9)	124
Kenya	50.6 (50.0–56.1)	311	36.1 (28.1–44.0)	145
Liberia	61.3 (58.1–64.5)	895	69.9 (66.0–73.8)	539
Madagascar	42.1 (34.8–49.4)	177	36.6 (25.9–4.3)	81
Malawi	73.3 (67.4–79.3)	216	88.6 (83.6–93.7)	156
Mali^c^	n/a	n/a	n/a	n/a
Mozambique	74.4 (69.9–78.9)	366	69.2 (63.9–74.5)	292
Rwanda	70.8 (63.2–78.4)	140	98.0 (95.1–100.0)	101
Senegal	73.0 (67.0–80.3)	176	47.5 (38.8–56.2)	131
Tanzania mainland	68.9 (66.7–72.1)	785	99.6 (99.0–100.0)	548
Uganda	65.4 (63.2–67.5)	1849	71.8 (69.3–74.4)	1240
Zambia	53.4 (48.6–58.2)	417	32.2 (26.1–38.4)	225
Zanzibar	87.2 (76.7–97.8)	42	100.0	36

*95* *% CI* 95 % confidence interval, *N* number of children

^a^Although weighted proportions are presented, the N values are unweighted and therefore, may not match exactly

^b^Children who received only ACT are excluded because the database does not contain information on when they started the treatment

^c^No information on time to treatment

Region of residence or type of place of residence were significantly associated with access to prompt treatment with any anti-malarial medicine in ten countries, making geography the most frequent association. In Angola, Benin, and Uganda, urban residence was significantly associated with prompt treatment (p value = 0.042, p value <0.0001, and p value = 0.039, respectively), whereas in Liberia, more rural children received prompt treatment (p value = 0.025).

Wealth quintile was significantly associated with prompt treatment in six countries. In most of these countries, children in the two highest wealth quintiles were most likely to receive prompt treatment. In contrast, children in the lowest wealth quintile in Angola and Liberia were most likely to have received prompt treatment (p value = 0.030 and p value <0.0001, respectively).

Maternal characteristics were associated with prompt treatment with any anti-malarial medicine in some countries. In general, the percentage of children who received prompt treatment was high among children whose mothers had at least a secondary education. In Kenya and Mozambique, prompt access to treatment with any anti-malarial medicine was significantly lower among children with mothers aged 15–19 years and higher among those with mothers aged 20–29 years (p value = 0.043 and p value < 0.0001, respectively).

Children’s demographic characteristics were less likely to be significantly associated with prompt access to anti-malarial treatment. In Ghana, more male children received prompt treatment than female children (p value = 0.049). In contrast, more female children in Senegal received prompt treatment compared to males (p value = 0.046). In Tanzania mainland, the percentage of children who received prompt treatment was significantly higher among children ages 24–59 months compared to younger children (p value = 0.056) (Table 4).

**Table 4:** Socio-economic and demographic characteristics significantly associated with access to prompt treatment with any anti-malarial drug among children under five

Country	Variable significantly associated with prompt treatment with any anti-malarial drug		Category with highest percentage of children who received prompt treatment with any anti-malarial drug	
	Variable	*p* value	Category	% (95% CI)
A	B	C	D	E
Angola	Relationship to the head of household	0.032	Grandchild	64.2 (55.3–73.2)
	Education level of the mother	0.029	Secondary and above	68.1 (60.7–75.6)
	Region of residence (endemicity)	0.032	Mesoendemica estavel	67.4 (61.0–75.6)
	Place of residence	0.042	Urban	62.6 (57.9–67.3)
	Wealth quintiles	0.030	Lowest	67.5 (54.3–80.6)
Benin	Education level of the mother	<0.0001	Secondary and above	85.2 (79.4–91.0)
	Region of residence	<0.0001	Littoral	91.0 (85.6–96.1)
	Place of residence	<0.0001	Urban	84.5 (81.9–87.1)
	Wealth quintiles	<0.0001	Highest	84.1 (80.0–88.1)
Ethiopia^a^	n/a	n/a	n/a	n/a
Ghana	Child’s sex	0.049	Male	59.4 (50.5–68.3)
	Region of residence	<0.0001	Upper east	87.0 (72.9–100.0)
Kenya	Age of the mother	0.043	20–29 years	53.0 (45.3–60.6)
	Education level of the mother	0.008	Secondary and above	66.5 (54.3–78.7)
	Region of residence	<0.0001	Central province	86.0 (63.0–100.0)
	Wealth quintiles	0.023	Fourth	65.5 (51.4–79.6)
Liberia	Place of residence	0.025	Rural	65.6 (61.0–70.0)
	Wealth quintiles	<0.0001	Lowest	69.8 (63.6–75.9)
Madagascar	Region of residence	<0.0001	n/a^b^	n/a
Malawi	Relationship to the head of household	0.033	Child	75.5 (69.4–81.6)
Mali^a^	n/a	n/a	n/a	n/a
Mozambique	Age of the mother	<0.0001	20–29 years	79.1 (73.1–85.1)
	Region of residence	<0.0001	Tete	99.7 (74.3–100.0)
Rwanda	Wealth quintiles	0.051^c^	Highest	81.1 (64.6–97.6)
Senegal	Child’s sex	0.046	Female	84.0 (75.1–92.9)
Tanzania mainland	Child’s age	0.056^c^	24–59 months	73.0 (68.0–77.0)
	Region of residence	<0.0001	Mtwara	96.4 (89.6–100.0)
Uganda	Region of residence	<0.0001	Karamoja	81.1 (75.0–87.1)
	Place of residence	0.039	Urban	68.6 (63.6–73.6)
Zambia	Education level of the mother	0.007	Secondary and above	65.7 (56.2–75.2)
	Region of residence	<0.0001	Southern	78.9 (66.5–91.3)
	Wealth quintiles	<0.0001	Fourth	66.8 (56.9–76.8)
Zanzibar^a^	n/a	n/a	n/a	n/a

*p* values (column C) indicate that the corresponding variable (column B) is significantly associated with prompt treatment with any anti-malarial drug; categories in column D are those that have the highest proportion of children who received prompt treatment with any anti-malarial drug for each corresponding variable in column B

*95* *% CI* 95 % confidence interval, *N* number of children

^a^Data on time to treatment was not available

^b^Region of residence is significantly associated with prompt treatment with any anti-malarial drug; however, the number of children for each of the 20 regions is fewer than 30, so percentages were not computed

^c^Although these values are p > 0.05, they were included because they show borderline significance

### Profiling children under five who received prompt treatment with ACT

To identify the socio-economic profile of children who received prompt and effective treatment, data from all children who received any anti-malarial drug across the 13 countries were pooled. Eight independent variables were examined, but only five were included in the final model. The model comprised 20 nodes, of which 13 were terminal nodes. Parent nodes included at least 100 children, whereas child nodes included at least 50 children.

**Fig. 2:**
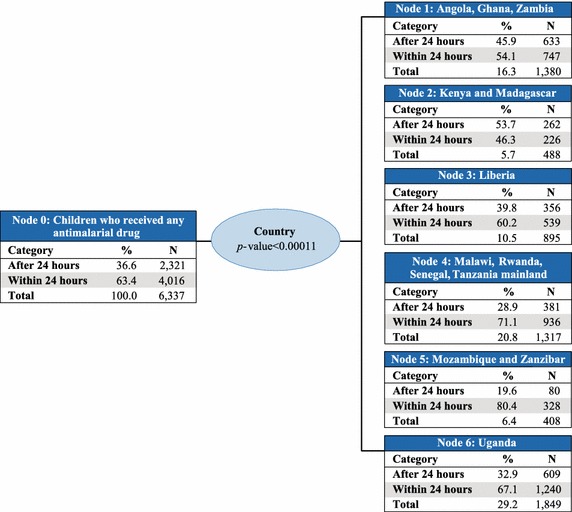
Prompt treatment with ACT among children under five treated with any anti-malarial medicine—Chi square automatic interaction detector tree diagram, level 1. *N* number of children. The *total line* of each *box* represents the share of the total number of children who received any anti-malarial treatment

**Fig. 3:**
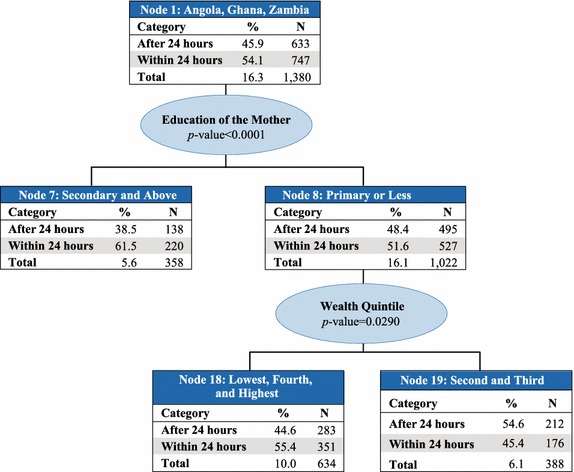
Node 1—Angola, Ghana, and Zambia. *N* number of children. The *total line* of each *box* represents the share of the total number of children who received any anti-malarial treatment

**Fig. 4:**
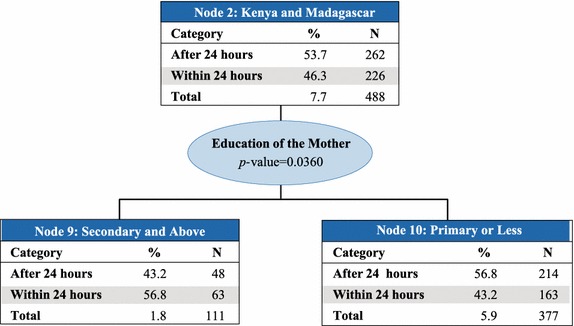
Node 2—Kenya and Madagascar. *N* number of children. The *total line* of each *box* represents the share of the total number of children who received any anti-malarial treatment

**Fig. 5:**
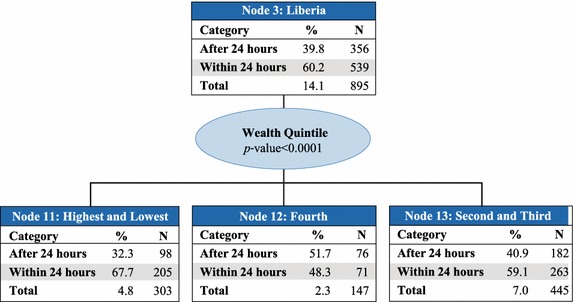
Node 3—Liberia. *N* number of children. The *total line* of each *box* represents the share of the total number of children who received any anti-malarial treatment

**Fig. 6:**
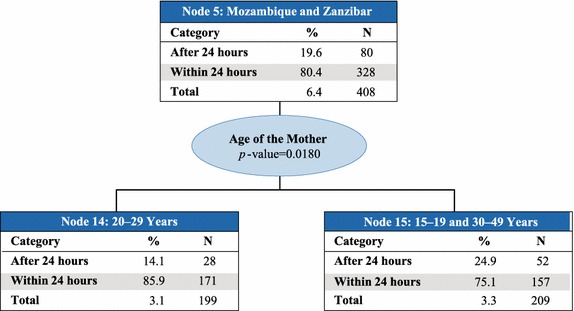
Node 5—Mozambique and Zanzibar. *N* number of children. The *total line* of each *box* represents the share of the total number of children who received any anti-malarial treatment

**Fig. 7:**
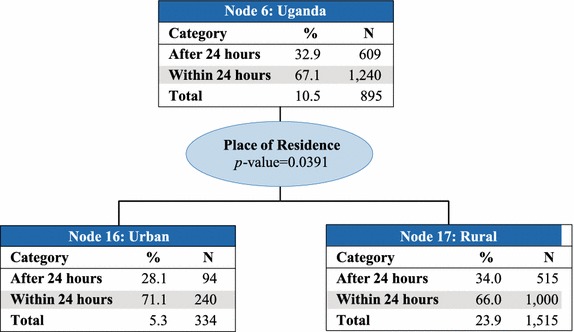
Node 6—Uganda. *N* number of children. The *total line* of each *box* represents the share of the total number of children who received any anti-malarial treatment

Country was the best predictor of access to prompt treatment with ACT (p value <0.0001) and therefore makes up the first level of the CHAID tree diagram. The analysis classified countries into six parent nodes that minimize variance of prompt and effective treatment within each node and maximize variance across nodes: (1) Angola, Ghana, and Zambia; (2) Kenya and Madagascar; (3) Liberia; (4) Malawi, Rwanda, Senegal, and Tanzania mainland; (5) Mozambique and Zanzibar; and, (6) Uganda. At the next levels of the CHAID tree diagram, these parent nodes were further split into child nodes by secondary and tertiary predictor variables, which were most commonly education of the mother or wealth quintile (Figs. 2, 3, 4, 5, 6, 7).

In Node 1–Angola, Ghana, and Zambia (Fig. 3), education level of the mother was the best predictor of prompt treatment with ACT (p value = 0.009). Among children whose mothers had secondary education or higher (Node 7), 61.5 % received prompt treatment with ACT. Among children whose mothers had primary education or less (Node 8), 51.6 % received prompt treatment with ACT. Household wealth quintile was also a predictor variable in Node 8 (p value = 0.029), with 55.4 % of children in the lowest, fourth, and highest wealth quintiles (Node 18) receiving prompt treatment with ACT, compared to only 45.4 % of their counterparts in the second and third quintiles (Node 19).

Education level of the mother was also the best predictor in Node 2–Kenya and Madagascar (Fig. 4) (p value = 0.036), with 56.8 % of children whose mothers had at least a secondary education (Node 9) receiving prompt treatment with ACT, compared to just 43.2 % of children whose mothers had primary education or less (Node 10). About 7.7 % of children who received any anti-malarial treatment live in these two countries, 46.3 % of whom received prompt treatment with ACT.

For children in Node 3–Liberia (Fig. 5), household wealth quintile was the best predictor of prompt treatment with ACT (p value <0.0001). About 67.7 % of children from the highest and lowest quintiles (Node 11), 48.3 % of children from the fourth quintile (Node 12), and 59.1 % of children from the second and third quintiles (Node 13) received prompt treatment with ACT. Children in Liberia represent 14.1 % of those who received any anti-malarial treatment.

Country was the only predictor for children in Node 4–Malawi, Rwanda, Senegal, and Tanzania mainland (Fig. 2); therefore, Node 4 is terminal. This group represents 20.8 % of children who received treatment with any anti-malarial medicine. Among these children, 71.1 % received prompt treatment with ACT.

In Node 5–Mozambique and Zanzibar (Fig. 6), age of the mother was the best predictor of prompt treatment with ACT (p value <0.0001). The proportion of children who received prompt treatment with ACT was significantly higher (85.9 %) among those with mothers ages 20–29 years (Node 14) compared to their younger and older counterparts in Node 15 (75.1 %, p value = 0.018). Children living in Mozambique and Zanzibar comprised just 6.4 % of children who received any anti-malarial treatment.

Place of residence was the best predictor in Node 6–Uganda (Fig. 7), with 71.1 % of children receiving prompt treatment with ACT in urban areas (Node 16) compared to 66.0 % in rural areas (Node 17) (p value = 0.039). Ugandans made up 10.5 % of all children who were treated with any anti-malarial medicine; 67.1 % of these children received prompt treatment with ACT.

### Chi square automatic interaction detector gain index

The gain index from the CHAID analysis can help highlight whether it is more efficient to target specific groups or to invest in the overall population, with potential trickle-down effects to those groups. The 13 terminal nodes of the CHAID tree diagram represent 13 homogenous sub-groups of children who received any anti-malarial treatment, grouped into three major clusters (Table 5).

**Table 5:** Chi square automatic interaction detector gain index

Cluster node	Node description	Node		Gain		% prompt^e^	Index^f^
		N^a^	%^b^	N^c^	%^d^		
A	B	C	D	E	F	G	H
Cluster 1		2059	32.5	1504	37.5	73.0	115.3
14	Mozambique and Zanzibar: children from 20 to 29 years old mothers	199	3.1	171	4.3	85.9	135.6
15	Mozambique and Zanzibar: children from 15 to 19 years old mothers and 30–49 years old mothers	209	3.3	157	3.9	75.1	118.5
16	Uganda: children from urban areas	334	5.3	240	6.0	71.9	113.4
4	Malawi, Rwanda, Senegal, and Tanzania: all children	1317	20.8	936	23.3	71.1	112.1
Cluster 2		3366	53.1	2102	52.3	62.4	98.5
11	Liberia: children from lowest and highest wealth quintiles	303	4.8	205	5.1	67.7	106.8
17	Uganda: children from rural areas	1515	23.9	1000	24.9	66.0	104.2
7	Angola, Ghana, and Zambia: children from mothers with secondary education and above	358	5.6	220	5.5	61.5	97.0
13	Liberia: children from second and third quintiles	445	7.0	263	6.5	59.1	93.3
9	Kenya and Madagascar: children from mothers with secondary education and above	111	1.8	63	1.6	56.8	89.6
18	Angola, Ghana, and Zambia: children from mothers with primary or no education; children from lowest, fourth, and highest wealth quintiles	634	10.0	351	8.7	55.4	87.4
Cluster 3		912	14.4	410	10.2	45.0	70.9
12	Liberia: children from the fourth wealth quintile	147	2.3	71	1.8	48.3	76.2
19	Angola, Ghana, and Zambia: children from mothers with primary or no education; children from second and third wealth quintiles	388	6.1	176	4.4	45.4	71.6
10	Kenya and Madagascar: children from mothers with primary or no education	377	5.9	163	4.1	43.2	68.2
Total		6337	100	4016	100	63.4	n/a

*N* number of children

^a^Number of children who received any anti-malarial treatment per node (demographic size in the sample)

^b^Demographic size in percentage = (0.1/Σ0.1) × 100

^c^Number of children who received prompt treatment with ACT

^d^Demographic size among children who received prompt treatment with ACT in percentage = (0.3/Σ0.3) × 100

^e^Proportion of children who received prompt treatment with ACT out of those who received any anti-malarial medicine = (0.3/Σ0.1) × 100

^f^Node index = [(0.3/Σ3)/(0.1/Σ0.1)] × 100

*Cluster 1* comprises children from urban areas in Uganda plus all children in Mozambique, Zanzibar, Malawi, Rwanda, Senegal, and Tanzania. This cluster represents 32.5 % of children who received treatment with any anti-malarial medicine, among which 73.0 % of children received prompt treatment with ACT. This group accounts for 37.5 % of all children who received prompt treatment with ACT.

*Cluster 2* includes children in all but the fourth wealth quintile in Liberia; children from rural areas in Uganda; children from mothers with a secondary education and above in Angola, Ghana, Zambia, Kenya, and Madagascar; and children from the lowest, fourth, and highest wealth quintiles in Angola, Ghana, and Zambia. This cluster accounts for 53.1 % of children who received treatment with any anti-malarial medicine, among which 62.4 % of children received prompt treatment with ACT. This group accounts for 52.3 % of all children who received prompt treatment with ACT.

*Cluster 3* includes children from the fourth wealth quintile in Liberia; children from mothers with primary or no education in Angola, Ghana, Zambia, Kenya, and Madagascar; and children from the second and third wealth quintiles in Angola, Ghana, and Zambia. This cluster represents 14.4 % of children who received treatment with any anti-malarial medicine. In this category, 45.0 % of children received prompt treatment with ACT, representing 10.2 % of all children who received prompt treatment with ACT (Table 5).

## Discussion

This study presented coverage of prompt treatment with ACT among febrile children under five years of age and also profiled those children who received this recommended treatment in selected countries in SSA. Most countries with high coverage of treatment with any anti-malarial medicine also had high coverage of treatment with ACT, perhaps since one is dependent on the other. Mali and Benin both depart from this trend; treatment with ACT was much less likely than treatment with other anti-malarial drugs. This may be due to ACT holding a particularly small share of the market compared to other anti-malarial drugs [18] or the fact that ACT policy was implemented only shortly before the surveys were conducted [1]. These countries show how important policy and markets are to ensuring access to prompt and effective treatment for malaria.

The cross-country CHAID analysis revealed that the country of residence is the best predictor of prompt and effective treatment, followed by maternal education, household wealth quintile, maternal age, and place of residence. It is perhaps unsurprising that country is the best predictor of prompt and effective treatment since malaria treatment policies differ among countries and are in various stages of implementation (Table 1). National drug regulations also affect the availability of anti-malarial drugs, including ACT, and may therefore also affect the uptake of prompt and effective treatment. This analysis highlights the importance of ensuring that first-line drug policies are synchronized with latest epidemiological information and are accompanied by efficient rollout and awareness raising campaigns. Results related to country of residence may also be a reflection of malaria transmission and epidemiology. For example, Node 2 comprises Kenya and Madagascar, which are the only two countries in the study with a majority of the population living outside of high-transmission areas [1]. Uganda and Liberia, which have the highest parasite prevalence of the countries in the study, each made up their own node.

Children were more likely to have received prompt and effective treatment if their mothers had at least a secondary education (Angola, Ghana, Zambia, Kenya, and Madagascar). With the exception of Angola, these countries also have higher than average proportions of women with at least a secondary education. Similar findings were reported by a study in which febrile children of educated women were more likely to receive a malaria test than children of non-educated women [19]. Furthermore, there is substantial literature showing positive correlations between children’s uptake of health services and mother’s level of education, independent of other social and economic factors [20, 21]. Educated mothers may be more open to advances in public health and medicine, more aware of malaria symptoms and related health risks, more trusting of providers, or more aware and better able to negotiate first-line services and treatment [22]. These characteristics may facilitate quick decision-making and therefore increase likelihood of prompt treatment. In SSA, women’s educational attainment is ever increasing, which suggests that the trend of accelerating gains in children’s health is also likely to continue [23].

In Mozambique and Zanzibar, children whose mothers were in their twenties were more likely to receive prompt and effective treatment than children whose mothers were older or younger. This is consistent with literature that suggests that adolescents are less likely to access maternal and child health services [24, 25]. Older mothers may be less likely to seek prompt and effective treatment if they are caring for multiple children. If they draw upon prior experience with febrile children, they also may be less inclined to follow new health messaging, thereby inhibiting prompt treatment. Further research on the relationships among mother’s age and education and prompt and effective treatment may help ensure that the mothers least likely to seek health services can and will access timely malaria treatment for their children.

In Angola, Ghana, Zambia, and Liberia, children from both the lowest and highest wealth quintiles were among those most likely to receive prompt and effective treatment. Children from the lowest wealth quintile may be most likely to take advantage of free ACT offered in the public sector in Angola, Zambia, and Liberia [1]. On the other hand, children from the highest wealth quintile may be more likely to seek care outside the public sector or may be able to afford first-line ACT from private retailers. Having access to these additional options for malaria care may facilitate prompt treatment, particularly if the locations are closer to home. This may change as community case management interventions improve access to prompt treatment by reducing the distance between febrile children and trained prescribers of ACT. Increasing coverage of malaria interventions has improved equity [26–28], so it is possible that further gains in coverage may cancel inequities both within and across countries. Targeted research on equity will help ensure universal access to prompt and effective treatment.

Availability of prompt and effective treatment or quality of care issues may also explain why place of residence was a secondary predictor of prompt and effective treatment in Uganda. Urban areas tend to have more options for health care (i.e., both public and private providers) and better access to preferred treatment compared to rural areas, which are mostly considered remote areas [29]. Affordable Medicines Facility-malaria evaluators reported higher availability of ACT in urban outlets in Uganda than rural areas, and even with an increase in availability at endline, rural areas remained behind [30]. This evidence supports the findings of the CHAID analysis, which showed that urban children in Uganda were more likely to receive prompt and effective treatment than rural children.

These are the predictors of which children are most likely to receive prompt and effective treatment, but a next step is strategizing for future interventions. The gain index from the CHAID analysis can help highlight whether it is more efficient to target specific groups or to invest in the overall population, with potential trickle-down effects to those groups. Increasing coverage in a particular node or cluster will yield corresponding returns at the population level, quantified by the gain index. For example, the gain index of Cluster 3 is just 70.9, showing that these children are much less likely to receive prompt and effective treatment than the overall population. Since Cluster 3 comprises only 14.4 % of children who received treatment with any anti-malarial medicine (i.e., this study’s target population), it is a relatively small population that may be easier to target with an intervention to increase access to prompt treatment. However, it may not be cost effective to invest significant resources into a small group like this because the benefits to national coverage goals may be limited. On the other hand, efforts to increase the proportion of children in Cluster 2 who receive prompt and effective treatment from 62.4 to 100 % may be more feasible since baseline coverage is higher. Investing in these children may also yield more visible public health impact since this cluster represents a majority (53.1 %) of the target population. These results may be useful in strategic planning for malaria control interventions at the national and subnational levels. Close monitoring can help malaria control programs determine if these children are being reached by future interventions in the quest for universal coverage.

Although this study presents informative findings, it does have limitations. It is difficult to track the extent to which patients with diagnosed malaria receive prompt and effective treatment using self-reported survey data because testing and treatment information are traditionally not linked in survey data. Self-reported survey data also require caretakers to identify specific drugs, which may be especially difficult in countries where many malaria drugs are on the market, compared to countries where only ACT is available [31]. Finally, timeliness data rely on the ability of caretakers to determine whether treatment commenced the same day or next day as the onset of fever, which can be difficult to ascertain.

In addition, data were collected from surveys during a 6-year span from 2006 to 2012. This time difference in survey implementation could affect comparability in pooled results, as could the type of survey conducted (DHS, MIS, or A&PS). Furthermore, varying amounts of time between malaria policy implementation and ACT rollout could differently affect coverage and access to prompt and effective treatment within countries and across countries. There is also considerable variation in the distribution of wealth quintiles and in equity of health interventions over time and among countries [32]. There is substantial subnational variation in malaria prevalence in several countries in SSA, which may affect expectations regarding ACT use and access to prompt treatment.

Finally, the data collected in these surveys do not examine additional factors related to access and social acceptability of malaria treatment and services. This information could help refine the profiles of children who received prompt and effective treatment. Future CHAID analyses could also include more complex covariates, such as source of treatment.

## Conclusion

This study reveals that country of residence, maternal education, place of residence, and socio-economic status are key predictors of prompt access to malaria treatment. The prominence of country as a predictor variable highlights the roles ACT policy implementation and rollout may play in access to prompt and effective treatment. The other key variables were of differing importance across countries, reminding implementers that understanding the context of their programmes is critical to success. Achieving universal coverage and the elimination agenda will require effective monitoring and early detection of coverage disparities within populations. Although national surveys provide a sporadic opportunity for this work, investing in routine surveillance will improve real-time tracking of ACT coverage and corresponding action, in line with recent recommendations from WHO’s Global Technical Strategy for Malaria.
